# CD4 Cell Count Trends after Commencement of Antiretroviral Therapy among HIV-Infected Patients in Tigray, Northern Ethiopia: A Retrospective Cross-Sectional Study

**DOI:** 10.1371/journal.pone.0122583

**Published:** 2015-03-27

**Authors:** Addisu Asfaw, Dagim Ali, Tadele Eticha, Adissu Alemayehu, Mussie Alemayehu, Filmon Kindeya

**Affiliations:** 1 Department of Pharmacy, College of Health Sciences, Mekelle University, Mekelle, Ethiopia; 2 Department of Public Health, College of Health Sciences, Mekelle University, Mekelle, Ethiopia; 3 Advocacy and Communication HIV Division, Social Mobilization, Tigray Regional Health Bureau, Mekelle, Ethiopia; Rush University, UNITED STATES

## Abstract

**Background:**

The rate and extent of CD4 cell recovery varies widely among HIV-infected patients with different baseline CD4 cell count strata. The objective of the study was to assess trends in CD4 cell counts in HIV-infected patients after initiation of antiretroviral therapy in Tigray, Northern Ethiopia.

**Methods:**

A retrospective cross-sectional study was conducted by reviewing medical records of HIV patients who received antiretroviral treatment at twenty health centers in Tigray region during 2008–2012. Multi-stage cluster sampling technique was employed to collect data, and the data were analyzed using SPSS version 20.0 software.

**Results:**

The median change from baseline to the most recent CD4 cell count was +292 cells/μl. By 5 years, the overall median (inter-quartile range, IQR) CD4 cell count was 444(263-557) cells/μl while the median (IQR) CD4 cell count was 342(246-580) cells/μl among patients with baseline CD4 cell counts ≤200 cells/μl, 500(241-557) cells/μl among those with baseline CD4 cell counts of 201–350 cells/μl, and 652(537-767) cells/μl among those with baseline CD4 cell counts >350 cells/μl. Higher baseline CD4 cell counts and being male were independently associated with the risk of immunological non-response at 12 months. Furthermore, it was also investigated that these factors were significant predictors of subsequent CD4 cell recovery.

**Conclusions:**

Patients with higher baseline CD4 cell stratum returned to normal CD4 Cell counts though they had an increased risk of immunological non-response at 12 months compared to those with the least baseline CD4 cell stratum. The findings suggest that consideration be given to initiation of HAART at a CD4 cell count >350 cells/μl to achieve better immune recovery, and to HIV-infected male patients to improve their health seeking behavior.

## Introduction

CD4 cell counts are commonly used markers of HIV disease progression and for starting and monitoring antiretroviral treatment in the absence of viral load [1,2]. Sustained increase in the CD4 cell response to highly active antiretroviral therapy (HAART) and suppression of HIV load were both associated with greater increases in CD4 cell counts [3]. Although an increase in CD4 cell count is achieved and maintained in all CD4 cell strata to six years, only patients with a baseline CD4 cell count of >350 cells/μl had CD4 cell counts that returned to nearly normal levels [4]. The World Health Organization (WHO) recommended ART should be initiated in HIV patients with a baseline CD4 cell count <350 cells/μl [5].

Commencement of ART at a baseline CD4 cell count <200 cells/μl may lead to a high proportion of patients with acquired immunodeficiency syndrome (AIDS) and a high case fatality rate [6,7]. A significant percentage of HIV-infected patients who initiated therapy with a CD4 cell count <200 cells/mm do not achieve a normal CD4 cell count, even after a decade of otherwise effective antiretroviral treatment [8]. In contrast, a study conducted among HIV-infected patients with very advanced immunodeficiency commencing antiretroviral treatment in sub-Saharan Africa investigated that patients with baseline CD4 counts <50 cells/μl had similar/higher rates of CD4 cell recovery and a lower risk of immunological non-response [9]. Another study from India demonstrated a high percentage of HIV patients with virologic and immunologic response after commencement of ART irrespective of the baseline CD4 cell count or viral load [10].

To the best of our knowledge, there are no studies from the study area concerning rates of CD4 cell recovery and rates of immunological non-response to ART among HIV-infected patients. Therefore, we conducted a retrospective study in order to assess the trends of CD4 cell recovery among HIV patients after initiation of antiretroviral therapy in Tigray, Northern Ethiopia.

## Methods

### Study setting and population

The study was conducted in Tigray region, Northern Ethiopia. Twenty health centers in the six administrative zones of the region were included in the study. A retrospective study was conducted by reviewing medical records of HIV patients aged 18 years or greater who received antiretroviral treatment at twenty health centers in Tigray region, Northern Ethiopia during 2008–2012. ART delivery was started in September 2003 as fee service and the free ART program started in March 2005. Pregnant women, seriously ill and diabetic patients were also excluded.

### Sample size and sampling

The required sample was calculated using a single population proportion formula. The following parameters were taken into account during the calculation of sample size: 50% rate of CD4 cell count recovery among HIV-infected patients (to achieve maximum representative sample size), 95% confidence interval and 5% margin of error. Then the determined sample size, after considering 10% non-response rate, was multiplied by 2 to consider the cluster effect and increase power. Thus, a required sample size of 844 medical records of HIV patients was required for this study.

Multistage cluster sampling technique was used to select the different health centers. Hence, twenty health centers were randomly selected proportionally among the sixty health centers addressed by MSH/USAID care and support programme from the six administrative zones found in Tigray region. Study participants from the selected health centers were chosen by systematic random sampling method.

### Data collection and analysis

A baseline and 6-monthly CD4 cell count, and basic information such as patients’ sex, age, weight and WHO clinical stage were collected from medical records.

Data were entered and analyzed using SPSS for Windows, version 20.0. The median (IQR) in the absolute CD4 cell count at baseline and every six months thereafter was determined. Changes in CD4 cell count every six months were also examined and stratified on the basis of baseline CD4 cell count (≤200, 201–350, and >350 cells/μl).

Categorical variables were summarized as frequencies and percentages while numerical variables with non-normal distribution were summarized as median and IQR. Logistic regression was conducted to examine factors associated with the risks of immunological non-response (defined as failure to attain an absolute CD4 cell count increase from baseline of at least 50 cells/μl at 12 months of ART). A multivariate least-squares linear regression analysis was employed to assess changes in CD4 cell count on the basis of baseline CD4 cell count and these other covariates. An initial analysis included all covariates. A final multivariate model was computed using a backward stepwise approach, retaining only those variables that were statistically significant. All tests of significance were two-sided, with p<0.05 indicating statistical significance.

### Ethical approval

The study was approved for ethical issues by the Health Research Ethics Review Committee of College of Health Sciences, Mekelle University. Data collection was conducted after further approval of the study by the Tigray regional health bureau and directors of each health centers. The purpose of the study was explained to the study participants and all study participants gave a written informed consent.

## Results

### Baseline characteristics of patients

A total of 841 medical records were reviewed; most (68.7%) of the patients were female. At baseline majority (79.5%) of the patients were less than 45 years old with a mean age (SD) of 36.34 (9.48) years. More than half (56.2%) of the patients were in WHO clinical stage III at baseline. About two-third (64.4%) of the patients were found to have baseline CD4 cell counts less than 200 cells/μl. The weight distribution shows that nearly three-forth (75.1%) of patients were found to have weight of 40–60 Kg (Table 1).

**Table 1:** Baseline characteristics of the patients.

**Baseline variables**		**Frequency**	**Percentage**
Gender	Female	578	68.7
	Male	263	31.3
Age (years)	<45	669	79.5
	≥45	172	20.5
	Mean (SD)	36.34 (9.48)	
WHO clinical stage	I	117	13.9
	II	184	21.9
	III	473	56.2
	IV	67	8.0
Baseline CD4 count	≤200	542	64.4
	201–350	179	21.3
	> 350	69	8.2
	Missing data	51	6.1
Weight (kg)	<40	159	18.9
	40–60	632	75.1
	>60	49	5.8

### CD4 cell count changes after initiating ART

**Fig 1:**
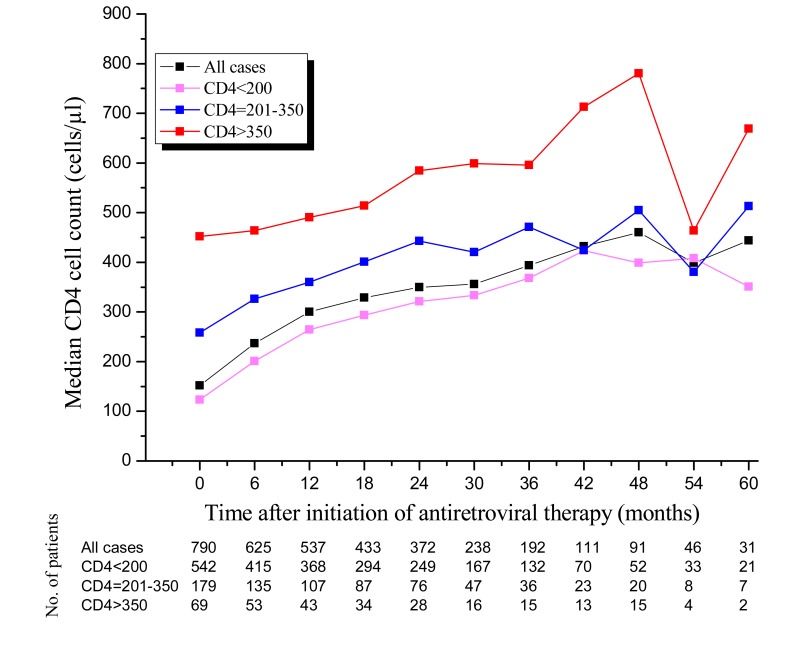
Median CD4 cell count after initiation of ART. Fig. 1 indicates the six monthly changes in the median CD4 cell count after the commencement of ART and the number of patients who had recorded CD4 data.

The six monthly changes in the median CD4 cell count after the commencement of ART and the number of patients having CD4 data is plotted in Fig. 1. The number of patients who had recorded CD4 data was decreased overtime. The overall median CD4 cell count had improved among HIV-infected patients over the five year period except in the 54^th^ and 60^th^ months where the median CD4 cell count showed a fall. The median change from baseline to the most recent CD4 cell count was +292 cells/μl, with the overall median (IQR) CD4 cell count increased to 444(263–557) cells/μl by 5 years after the start of ART. The overall median (IQR) CD4 cell count in our HIV patients increased by about 2-fold and 3-fold from baseline 152 (98–224) cells/μl, reaching 300(201–398) and 460(240–704) cells/μl at 12 and 48 months after initiation of ART, respectively. By 5 years, the median (IQR) CD4 cell count increased to 342(246–580) cells/μl among patients with a baseline CD4 cell count of ≤200 cells/μl, to 500(241–557) cells/μl among those with a baseline CD4 cell count of 201–350 cells/μl, and to 652(537–767) cells/μl among those with a baseline CD4 cell count of >350 cells/μl. The median (IQR) CD4 cell count of patients with a baseline CD4 cell count of ≤200 cells/μl were about doubled and tripled by 12 and 36 months after initiation of ART, respectively.

An analysis of the six monthly increase in CD4 cell count demonstrated that, the slope of the plotted overall CD4 cell count increased significantly (p<0.05, compared with the previous six months) until 48 months except between 18 and 30 months, where it slightly increased. The proportions of patients who achieved CD4 cell counts of ≥500 cells/μl after receiving 48 months of suppressive ART were 40.8%, 45% and 93.3% among patients with baseline CD4 cell counts of ≤200, 201–350 and >350 cells/μl, respectively. The percentages of patients who achieved CD4 cell counts of ≥750 cells/μl after receiving 48 months of ART were 19.8%, 25% and 60% among patients with baseline CD4 cell counts of ≤200, 201–350 and >350 cells/μl, respectively.

Baseline CD4 cell count was evaluated whether it was a risk factor associated with immunological non-response (defined as a CD4 cell increase of <50 cells/μl at 12 months). The percentages of patients failed to attain an absolute CD4 cell count increase from baseline CD4 cell count of at least 50 cells/μl at 12 months was 22.7%. The proportions of patients who were immunological non-responders were 17.9%, 28% and 50% among patients with baseline CD4 cell counts of ≤200, 201–350 and >350 cells/μl, respectively. Furthermore, logistic regression analysis revealed that baseline CD4 cell count was a significant predictor of immunologic failure. An increased risk of immunological non-response was independently associated with higher baseline CD4 cell counts of 201–350 (OR = 1.90, 95% CI: 1.14–3.18) and > 350 cells/μl (OR = 5.53, 95% CI: 2.77–11.04). An increment of <50 cells/μl was also independently associated with male patients. Male patients had about two times increased odds of risk of immunological non-response compared to female patients (OR = 2.02, 95% CI: 1.26–3.24) (Table 2).

**Table 2:** Results of logistic regression analysis of baseline characteristics associated with the risk of immunological non-response (an increase of <50 cells/μl).

**Characteristics**		**Risk of immunological non-response at 12 months**	
		**OR (95% CI)**	**p value**
Gender	Female	1	
	Male	2.02 (1.26, 3.24)	**0.004**
Age (years)	≤45	1	
	>45	1.34 (0.81, 2.22)	0.261
WHO clinical stage	I	1	
	II	1.07 (0.52, 2.17)	0.859
	III	1.21 (0.64, 2.31)	0.554
	IV	1.23 (0.47, 3.22)	0.678
Baseline CD4 count	≤200	1	
	201–350	1.90 (1.14, 3.18)	**0.014**
	>350	5.53 (2.77, 11.04)	**0.000**
Weight (kg)	<40	1	
	40–60	1.05 (0.50, 2.17)	0.901
	>60	1.26 (0.45, 3.55)	0.668

Multivariate regression analysis shown that baseline CD4 cell count was a significant determinant of subsequent CD4 cell recovery (Table 3). Two analyses are indicated; in the first, all covariates are included, whereas the second includes only those covariates that were statistically significant. In addition to baseline CD4 cell count, being male was associated with a less robust CD4 cell count response (p<0.05).

**Table 3:** Multivariate analysis of baseline characteristics associated with changes in CD4 cell count.

**Characteristics**	**Initial model**		**Final model**	
	Change in CD4 cell count, cells/μl	P value	Change in CD4 cell count, cells/μl	P value
Male sex	-76	0.003	-78	0.000
Age <45 years	55	0.069	-	
Baseline CD4 count, cells/μl				
≤200	Reference		Reference	
201–350	119	0.128	119	0.000
>350	258	0.001	251	0.000
WHO clinical stage				
I	Reference			
II	-2	0.914	-	
III	19	0.331	-	
IV	-29	0.257	-	
Weight (Kg)				
<40	Reference			
40–60	-16	0.458	-	
>60	-45	0.155	-	

**NOTE:** The initial model contained all covariates, and the final model contained only statistically significant covariates.

## Discussion

This retrospective study was carried out to assess the trends in CD4 cell recovery among HIV patients after initiation of ART and the effect of baseline characteristics on CD4 cell count response. Improvements in overall CD4 cell count among the patients was seen over time until year 4 though it declined thereafter. These findings are consistent with the retrospective longitudinal study conducted in eastern Ethiopia where the median CD4 lymphocyte count had improved over the five year period except at the 54^th^ and 60^th^ months where the median CD4 cell count showed a slight decline [11]. Other studies also clearly investigated that CD4 cell counts were increased significantly until year 4 though varied among different CD4 cell strata thereafter [4,8]. Actually HAART has been shown to improve survival rates in HIV-infected patients through its ability to increase the CD4 lymphocyte count in peripheral blood in addition to reducing HIV load to undetectable levels. This leads to a remarkable reduction in AIDS-related morbidity and mortality rates [12].

Immunological response rates were evaluated in patients with different baseline CD4 cell in this survey. These results indicated that patients with baseline CD4 cell counts of ≤200 cells/μ had greater rates of immunological response compared to those with higher baseline CD4 cell counts. These findings are in line with that of another study where patients with the lowest CD4 counts had similar or greater rates of CD4 cell recovery and a lower risk of immunological non-response [9]. However, in a study carried out in the EuroSIDA cohort, a lower CD4 cell count was associated with a lower rate of CD4 cell recovery at 12 months of HAART [13]. A retrospective study conducted by reviewing 459 medical records of adult treatment naive HIV patients reported that the outcomes of HIV patients did not differ by baseline CD4 cell count [14].

On the other hand, the present work reported that patients with lower baseline CD4 cell counts had lower peaks CD4 cell counts. Patients with a baseline CD4 cell count of >350 cells/μl stratum had CD4 cell counts that returned to almost normal levels. These results are consistent with that of a longitudinal observational study among patients receiving primary HIV care in Baltimore, Maryland which shows only patients with a baseline CD4 cell count of >350 cells/μl had CD4 cell counts that returned to nearly normal levels though CD4 cell count was increased and maintained in all CD4 cell count strata to 6 years [4]. Another study found that almost all patients who started therapy with a CD4 cell count ≥300 cells/mm were able to attain a CD4 cell count ≥500 cells/mm. A substantial proportion of patients who delay therapy until their CD4 cell count decreases to 200 cells/mm do not achieve a normal CD4 cell count, even after a decade of otherwise effective antiretroviral therapy [8]. Other studies also reported similar results that the CD4 cell count continued to increase through 18 months [15]. It was investigated that patients with a baseline CD4 cell count ≤200 cells/μl had the lowest peak CD4 cell count. Studies found that a lower CD4 cell count at the start of antiretroviral therapy was related to a lower plateau CD4 cell count [4,8]. Patients who started receiving HAART when their CD4 cell count was <350 cells/μl did not achieve a CD4 cell count that was as high as that achieved by other patients [16].

These findings indicate that the highest CD4 cell counts were achieved when HAART was started at a baseline CD4 cell count >350 cells/μl. The CD4 cell count is less likely to return to normal when HAART is started at lower CD4 cell counts, this could be a reason to consider initiating HAART before the CD4 cell count decreases to <350 cells/μl.

In this study, an increased risk of immunological non-response was significantly observed among male patients like another study [17]. Other studies also reported that the higher rate of subsequent CD4 cell recovery was observed among female patients than males [18,19]. In contrast, a study of immunological recovery in patients indicated that gender was not associated with the risk of immunological non-response [9]. Another study also investigated that gender was not associated with increases in CD4 cell counts [3]. The patients in the present study were predominantly female could reflect the feminization of the HIV epidemic, better health seeking behavior of women and possibly the linkage of treatment sites with the antenatal clinics and the prevention-of-mother-to-child HIV programs resulting in better immune recovery. Nevertheless, unimproved outcomes among male patients were because of poor health seeking behavior of men [20]. This includes lower rates of HIV testing, lower rates of repeat-testing and lower acceptance of linkage to HIV-care after a positive result. Men could improve decision-making not only for themselves, but likewise for the women and children who live in male dominated societies if they involve in health program [17]. Thus, males could require to be addressed by new strategies for HIV voluntary counseling and testing focused on workplace and leisure places usually seen by males, flexible clinic hours for working males in addition to more aggressive mobilization campaigns targeted to males.

In conclusion, the present findings confirm the previous studies that the degree of CD4 depletion prior to ART initiation is the most consistent determinant of subsequent immune reconstitution. Low baseline CD4 count at entry to an ART programme was associated with increased risks of morbidity and mortality [21]. A meta-analysis confirmed that there is a strong increased risk of death associated with initiating ART at lower CD4 counts over one year on ART in resource-limited settings [22]. Nevertheless, it has been shown that commencement of ART at higher CD4 counts is both clinically advantageous and leads to improved immune constitution [23]. Therefore, initiation of HAART before the CD4 cell count decreases to <350 cells/μl is recommended to achieve and maintain normal CD4 cell count. Better immune recovery among HIV-infected males could need improved health seeking behavior of men.
